# Supplementary material for: Ultrafast response of spontaneous photovoltaic effect in 3R-MoS2–based heterostructures
Source: Sci Adv. 2022 Dec 16;8(50):eade3759. doi: 10.1126/sciadv.ade3759 (PMC9757740; doi:10.1126/sciadv.ade3759)
Supplement: Supplementary file 1 — Figs. S1 to S14 Notes S1 to S7 References [file sciadv.ade3759_sm.pdf]

Science Advances
AAAS

Supplementary Materials for

Ultrafast response of spontaneous photovoltaic effect in 3R-MoS_2–based heterostructures

Jingda Wu *et al.*

Corresponding author: Ziliang Ye, zlye@phas.ubc.ca

Sci. Adv. **8**, eade3759 (2022)
DOI: 10.1126/sciadv.ade3759

This PDF file includes:

Figs. S1 to S14
Notes S1 to S7
References

Note S1. Power dependence of the autocorrelation signal

Note S2. Circuit model

Note S3. Temperature dependence of the shunt resistance

Note S4. Photocurrent temperature dependence

Note S5. IR-pulse induced photocurrent

Note S6. Symmetrized pump-probe signal and its comparison with the autocorrelation result

Note S7. Two-temperature model

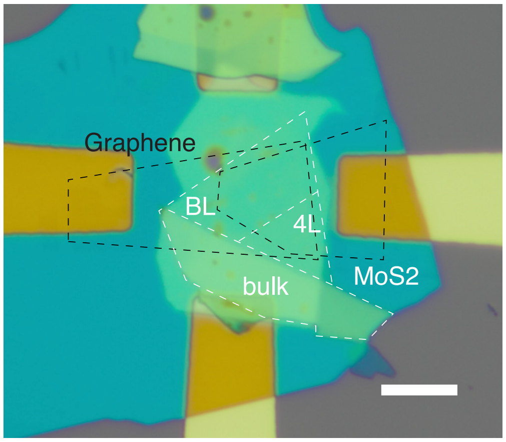

Fig. S1. Optical image of the device. The device region where graphene electrodes overlaps covers both bilayer and 4-layer MoS_2. Scale bar: 8 um.

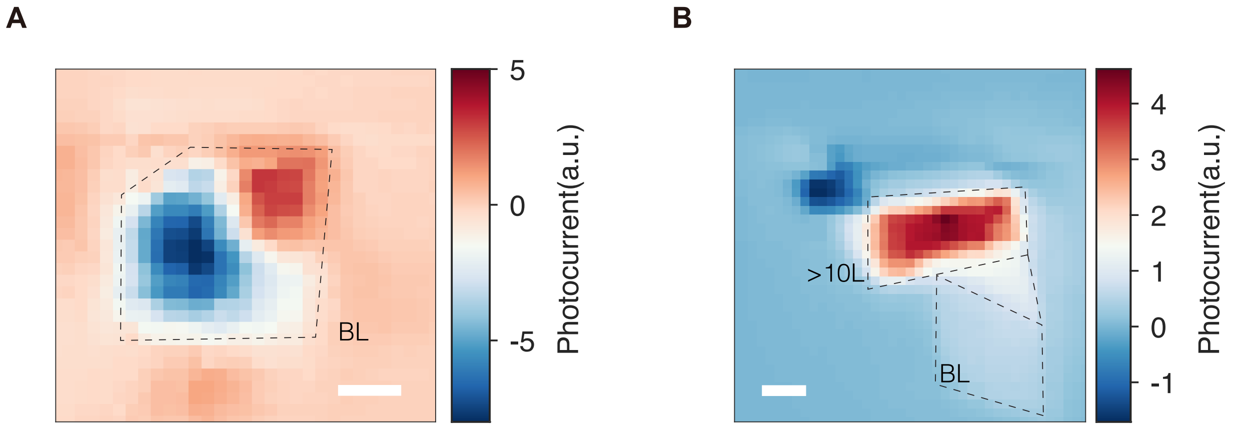

Fig. S2. Photocurrent mapping of additional devices. Photocurrent mapping from devices D2 (**A**) and D3 (**B**). D2 is purely BL and D3 has some regions with thick 3R-MoS_2. Opposite signs of photocurrent are due to domain difference (AB or BA stacking). Scale bar: 2 um.

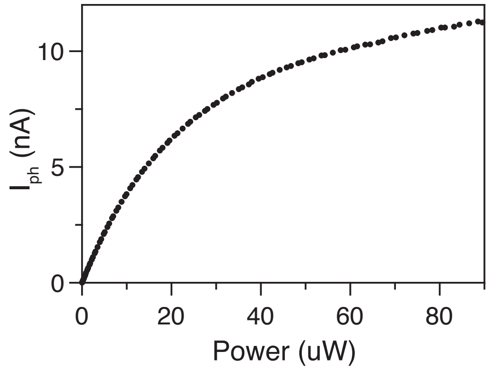

Fig. S3. Illumination power dependence of photocurrent. Photocurrent measured with above bandgap laser illumination (700 nm) at different powers, showing a sublinear power dependence.

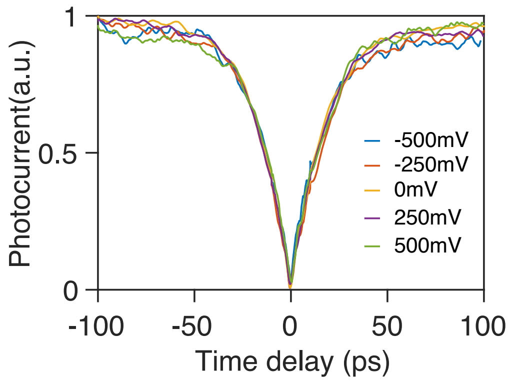

Fig. S4. Bias-dependent auto-correlation measurements on the BL region. Autocorrelation signals at the BL region measured at bias voltages from -0.5 V to 0.5 V. The raw data are normalized for comparison.

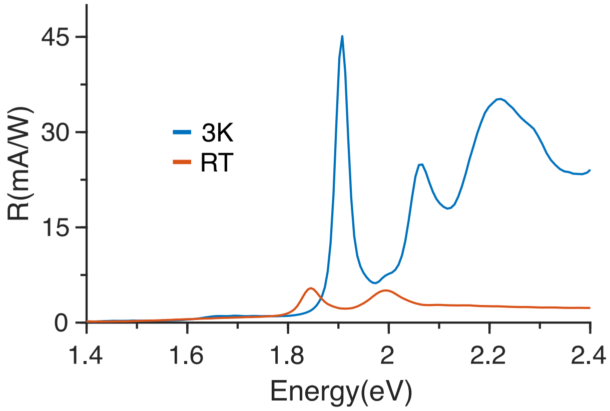

Fig. S5. Wavelength-dependent photoresponsivity of another BL device. The measurement is carried out for both RT and 3K, at around 0.5 uW for each wavelength to avoid saturation.

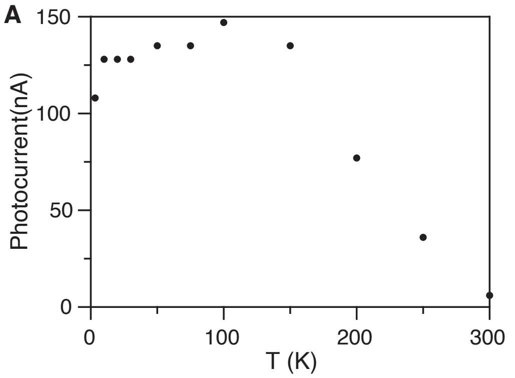

Fig. S6. Temperature dependence of photocurrent and resistance. **A**. The photocurrent under 20 uW 532 nm CW laser illumination from 3 K to room temperature. **B**. Total resistance of the device from 3K to room temperature.

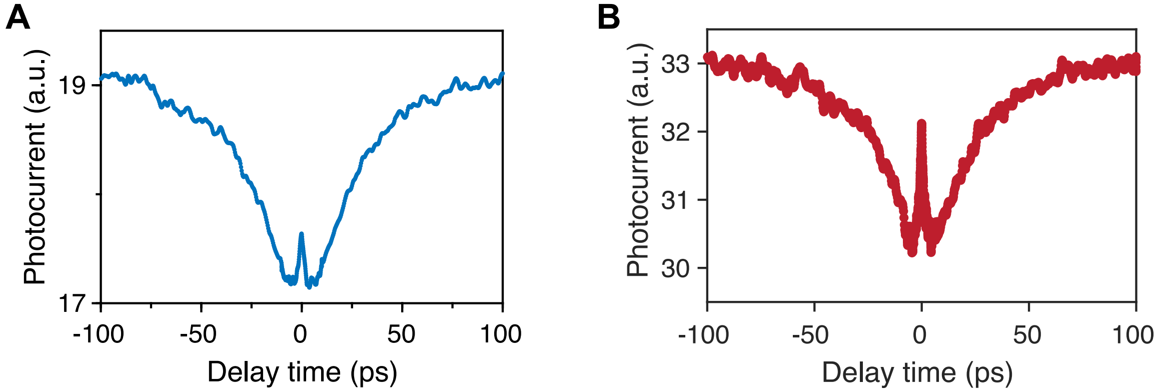

Fig. S7. Additional autocorrelation signals. (**A**) Autocorrelation signal from the 4L region on the device presented in the main text under 770 nm femtosecond pulse excitation. (**B**) Autocorrelation signal from a pure BL device under 800 nm femtosecond pulse excitation. The peaks at zero delay are from the PTE photocurrent saturation as discussed in the main text.

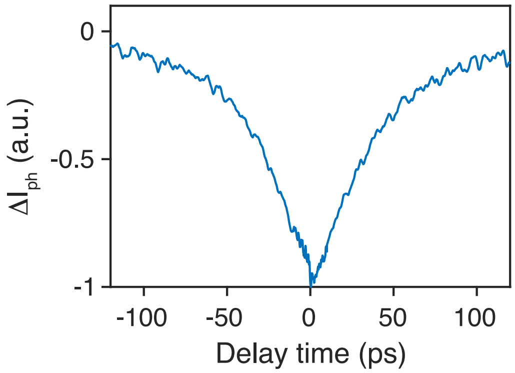

Fig. S8. Pump-probe photocurrent measurement of the 4L region at zero bias. The curve is asymmetric but the sharp drop is not as obvious as on BL region. The 20 ps scan around zero delay is performed at a finer temporal resolution than the rest.

Note S1. Power dependence of the autocorrelation signal

Previously, the photocurrent saturation in 2H-MoS_2 was shown to arise from an electronic origin. As a consequence, there is a significant power dependence in the time constant of photocurrent autocorrelation (*24*). In this work, we measure the autocorrelation signal at several different laser power levels (Fig. S8) and found the response time only slightly increases with increasing laser intensity, and it quickly saturates at above 100 uW of laser power at 76 MHz repetition rate and 50 fs pulse width. This behavior further confirms the thermal nature of the photocurrent saturation since the thermal conductivity, which determines the cooling dynamics and therefore recovery time of the device, does not change significantly above room temperature (*36*).

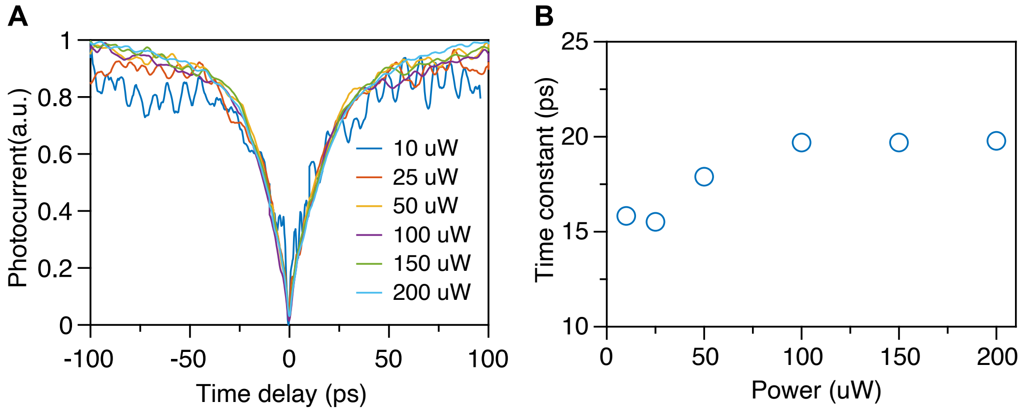

Fig. S9. Power-dependent autocorrelation measurements of BL region. (**a**) Autocorrelation of the BL region measured at different power levels. The signals are normalized for comparison. The extracted time constants are plotted in (**b**).

Note S2. Circuit model

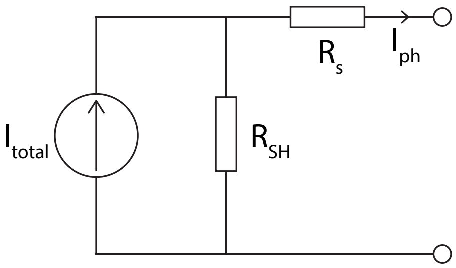

Fig. S10. Circuit model of the Gr/3R-MoS$_2$/Gr photodetector. I_{total} is the total photocurrent generated in the device, I_{ph} is the measured output current, R_{SH} is the shunt resistance of the device, R_s is the series resistance.

Fig. S10 shows an equivalent circuit model of the Gr/3R-MoS$_2$/Gr device. In the photovoltaic mode, the device is modelled as an ideal current source in parallel (series) with a shunt (series) resistor (*32, 43*). I_{total} is the total photocurrent generated in the device and I_{ph} is the measured output current. The shunt resistance R_{SH} mainly arises from the tunnelling or thermionic transport, with the latter dominating at above room temperature (*33*). R_s mainly consists of graphene contact resistance R_c. As a result, the measurable output photocurrent is

$I_{ph} = I_{total}\frac{R_{SH}}{R_{SH}+R_c}$. At room temperature, R_c is much larger than R_{SH} (*7*). Thus, we approximate the output current as

$$I_{ph} \approx I_{total}\frac{R_{SH}}{R_c}. \qquad (1)$$

Note S3. Temperature dependence of the shunt resistance

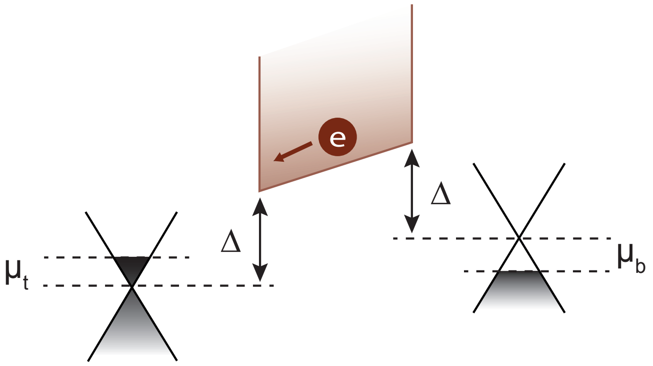

Fig. S11. Band alignment of Gr/3R-MoS$_2$/Gr junction. The Fermi-levels in the top and bottom graphene are aligned. The chemical potentials are denoted as μ_t and μ_b in top and bottom graphene, respectively.

Above room temperature, the shunt resistance of the device is dominated by over-the-barrier thermionic transport (*33*). The thermionic current from top graphene to bottom graphene can be written as

$$I_{therm} = A\frac{e}{h}\int_{\Delta}^{+\infty} D_t(E+\mu_t)D_b(E+\mu_b)[f_0(E-E_{ft}) - f_0(E-E_{fb})]dE. \qquad (2)$$

where A is a normalization factor, e is the charge of an electron and h is the Planck constant. Δ is the energy difference between the graphene Dirac point and the MoS$_2$ conduction band edge (Fig. S11). D_t and D_b are the density of states (DOS, $\frac{2|E|}{\pi(\hbar v_f)^2}$) in the top and bottom graphene, respectively. $f_0(E) = \frac{1}{\exp(\frac{E}{k_B T})+1}$ is the Fermi-Dirac distribution, while E_{ft} and

E_{fb} are the Fermi levels in the top and bottom graphene electrodes, respectively. At zero bias, $E_{ft} = E_{fb} = 0$. The chemical potentials relative to the Dirac points for the bottom and top layer graphene are denoted as μ_t and μ_b (Fig. S11), respectively. To derive the conductivity at zero bias, we assume there is a small bias δ between two electrodes, leading to a finite Fermi level difference $E_{ft} - E_{fb} = e\delta$. The zero bias conductivity is the limit of I/δ at $\delta \to 0$.

$$\lim_{\delta \to 0} \frac{I_{therm}}{\delta} = A\frac{e^2}{h} \int_{\Delta}^{+\infty} D_t(E+\mu_t)D_b(E+\mu_b)(-\frac{\partial f_0}{\partial E})dE, \tag{3}$$

For the case where $\Delta >> k_BT$, we can approximate the Fermi-Dirac distribution as the Boltzmann distribution, and $-\frac{\partial f_0}{\partial E} = \frac{1}{k_BT}\exp(\frac{-E}{k_BT})$. Thus we obtain

$$\sigma = 4A\frac{e^2}{h}\frac{1}{\pi^2(hv_f)^4}\int_{\Delta}^{+\infty}(E+\mu_t)(E+\mu_b)\frac{1}{k_BT}\exp(-\frac{E}{k_BT})dE, \tag{4}$$

$$\approx 4A\frac{e^2}{h}\frac{1}{\pi^2(hv_f)^4}\int_{\Delta}^{+\infty}E^2\frac{1}{k_BT}\exp(-\frac{E}{k_BT})dE, \tag{5}$$

$$= 4A\frac{e^2}{h}\frac{k_B^2T^2}{\pi^2(hv_f)^4}((\frac{\Delta}{k_BT})^2 + 2\frac{\Delta}{k_BT} + 2)\exp(-\frac{\Delta}{k_BT}), \tag{6}$$

The chemical potentials in the top and bottom graphene are negligible compared to Δ. Since $\Delta >> k_BT$, the inequality $(\frac{\Delta}{k_BT})^2 >> \frac{\Delta}{k_BT} >> 1$ also holds. Hence, we can further simplify equation (6) as

$$\sigma \approx \sigma_0 \exp(-\frac{\Delta}{k_BT}), \tag{7}$$

where $\sigma_0 = \frac{4A\Delta^2}{\pi^2(hv_f)^4}\frac{e^2}{h}$ is a constant. The shunt resistance is the inverse of conductance, yielding

$$R_{SH} \propto 1/\sigma \propto \exp(\frac{\Delta}{k_BT}). \tag{8}$$

Note S4. Photocurrent temperature dependence

Combining equation (1) and (8), we have

$$I_{ph} \propto \exp(\frac{\Delta}{k_B T}). \tag{9}$$

By fitting the photocurrent temperature dependence shown in the inset of Fig. 3c in the main text with equation (9), we extract Δ = 0.14 eV.

From equation (9), we can also derive the photocurrent I'_{ph} at a device temperature T_d with respect to the photocurrent I' at the ambient temperature T_0.

$$I'_{ph} = I' \exp(\frac{\Delta}{k_B}(\frac{1}{T_d} - \frac{1}{T_0})). \tag{10}$$

We can then assign $\alpha = \exp(\frac{\Delta}{k_B}(\frac{1}{T_d} - \frac{1}{T_0}))$ as the dimensionless photocurrent saturation factor mentioned in the main text.

Note S5. IR-pulse induced photocurrent

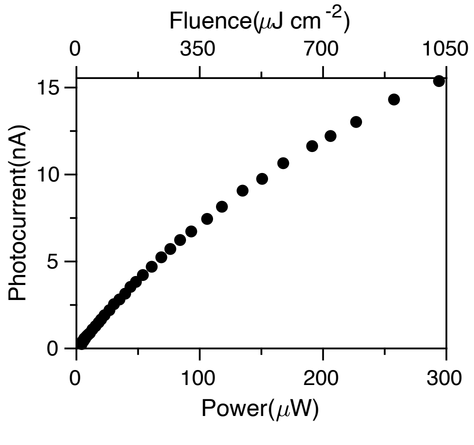

Fig. S12. Photocurrent power dependence under IR-pulse excitation. The photocurrent excited by 1030-nm femtosecond pulse at different power levels.

In the non-degenerate pump-probe photocurrent measurement, the IR-pulse also generates a finite photocurrent (Fig. S12). Such a photocurrent could have multiple extrinsic origins such as a) hot-carrier injection from graphene (*25*), b) two-photon excitation of photocarriers,

c) excitation of MoS2 mid-gap defect states (*30, 32, 39*). For case a) and b), the photocurrent should have a linear or super-linear temperature/power dependence, while for case c), the photocurrent should have a similar saturation behavior as above-bandgap excitation. Our measured IR-photocurrent power dependence favors the last mechanism.

With the assumption of defect-related excitation, the IR-pulse-induced photocurrents are modulated according to the same circuit model as those induced by the VIS-pulse. As a result, we can apply the same correlation formula to both photocurrents, with the temperature increase generated by the opposite pulse, namely

$$\bar{I}_{IR}(\Delta\tau) = \frac{1}{\tau_{rep}} \int_0^{\tau_{rep}} \alpha_{VIS}(t) I_{IR}(t + \Delta\tau) dt, \tag{11}$$

and

$$\bar{I}_{VIS}(\Delta\tau) = \frac{1}{\tau_{rep}} \int_0^{\tau_{rep}} \alpha_{IR}(t) I_{VIS}(t + \Delta\tau) dt. \tag{12}$$

In our experimental configuration, the IR-pulse (350 uJ cm^{-2}) generates two orders of magnitude more heating and approximately an order of magnitude more photocurrent than the VIS-pulse (2 uJ cm^{-2}). Thus, the maximum change in $\bar{I}_{IR}$ is more than one order of magnitude smaller than that in $\bar{I}_{VIS}$. As a result, we conclude the IR-pulse induced photocurrent does not affect our main result and is neglected in the data analysis.

Note S6. Symmetrized pump-probe signal and its comparison with the autocorrelation result.

As mentioned in the main text, the signal measured by the non-degenerate pump-probe photocurrent spectroscopy can be expressed as a cross-correlation between the photocurrent generated by the visible beam and heat generated by the IR beam, which is

$$\bar{I}(\Delta\tau) = \frac{1}{\tau_{rep}} \int_0^{\tau_{rep}} \alpha_{IR}(t) I_{VIS}(t + \Delta\tau) dt, \tag{13}$$

or in a compact form

$$\bar{I}(\Delta\tau) \propto \alpha_{IR} \star I_{VIS}, \tag{14}$$

where subscripts denote the pulses with which the physical processes are associated.

On the other hand, since the two beams are symmetric in the autocorrelation experiment, each beam can cause a photo-thermal saturation effect on the photocurrent generated by the other beam. As a result, the autocorrelation signal can be expressed as the sum of two contributions.

$$\bar{I}(\Delta\tau) \propto \alpha_1 \star I_2 + \alpha_2 \star I_1, \tag{15}$$

where subscripts refer to the beam index. With the two beams being identical but symmetric in time, we can rewrite the expression as

$$\bar{I}(\Delta\tau) \propto (\alpha_1 \star I_2)(\Delta\tau) + (\alpha_1 \star I_2)(-\Delta\tau). \tag{16}$$

The second term is the time-reversed copy of the first term, suggesting the autocorrelation signal can be reproduced by symmetrizing the pump-probe signal if one can distinguish the effect of individual beams. In the following, we apply such a symmetrization procedure to the non-degenerate pump-probe photocurrent signal:

$$\bar{I}(\Delta\tau) \propto (\alpha_{IR} \star I_{VIS})(\Delta\tau) + (\alpha_{IR} \star I_{VIS})(-\Delta\tau). \tag{17}$$

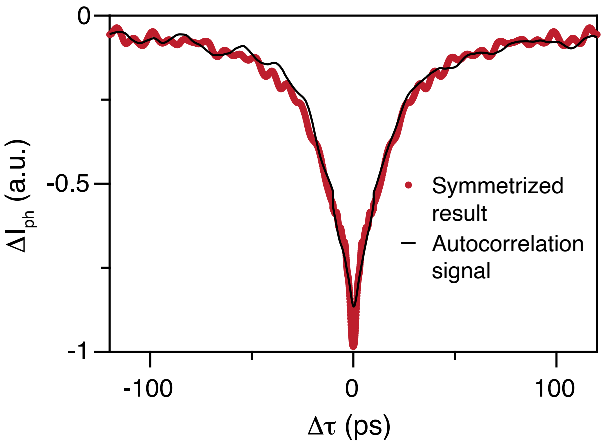

Fig. S13. Comparison of symmetrized pump-probe and autocorrelation photocurrent signals. According to Eq. (17), the symmetrization is done by inverting the time axis of the pump-probe photocurrent data (Fig. 3b) and adding it back to the original one. The autocorrelation signal is the same as shown in Fig. 1d.

As shown above, the symmetrized pump-probe photocurrent signal shares similar dynamics with the autocorrelation data collected in the same device at the same temperature, which justifies our simple model. Since the decay related to τ_1 is much faster than the thermal decay, the pump-probe photocurrent result is asymmetric and the signal level at negative delay is much smaller than that at positive delay, given the same interval from zero delay. Therefore, the agreement between the two plots confirms the slow decay in the autocorrelation experiment is mainly contributed by the heat dissipation process.

On a finer scale, there exists a mismatch between the two near zero delay, where the symmetrized signal has a sharper dip related to the ultrafast photocurrent response. We attribute the difference to the photo-thermoelectric (PTE) effect in the graphene electrodes, as discussed in the main text. When an ultrafast pulse heats up the electrons in graphene, the PTE effect induced by the other pulse is reduced, and thus causing a sharp feature with a positive sign. The magnitude of the PTE peak is highly device and temperature dependent, and usually dominates the signal near zero delay in the autocorrelation experiment (Fig. 2b and Fig. S6). In contrast,

the probe beam in the pump-probe photocurrent experiment is very weak and causes negligible heating, thus avoiding the interference from the PTE effect. This comparison underscores the benefit of the non-degenerate pump-probe photocurrent spectroscopy technique we developed.

Note S7. Two-temperature model

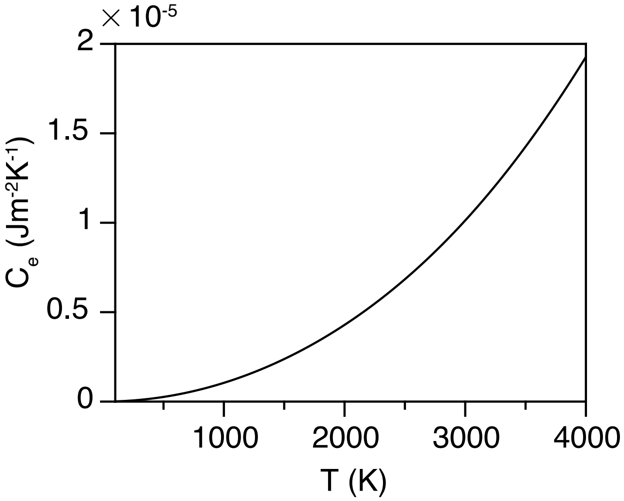

Fig. S14. Graphene electronic heat capacity. The values are calculated from the tight-binding model.

The temperature evolution is modelled through a two-temperature model consisting of the graphene electronic temperature T_e and device temperature T_d, which represents graphene and MoS_2 lattice temperature. After graphene is excited by intense pulses, the electronic temperature rapidly cools through supercollision with acoustic phonons (*27, 28, 38*), with the interaction term $\Gamma_{e-ph} = A(T_e^3 - T_l^3)$, where $A = 0.75\,\mathrm{W\,m^{-2}\,K^{-3}}$ is the supercollision coupling constant (*28*) and T_l is the graphene lattice temperature. With only four atomically thin layers in a symmetric stacking order, graphene quickly reaches thermal equilibrium with MoS_2, and both contribute to the change in the shunt resistance. Therefore, we can use the device temperature T_d to capture the photocurrent dynamics. The whole device cools through the thick hBN encap-

sulation, which is approximately at room temperature T_0. We may then write the rate equations as

$$\frac{C_e \partial T_e}{\partial t} = - A(T_e^3 - T_d^3) + Q_{IR}(t), \tag{18}$$

$$\frac{C_d \partial T_d}{\partial t} = A(T_e^3 - T_d^3) - G_{G-hBN}(T_d - T_0). \tag{19}$$

C_e is the graphene electronic heat capacity, which can be calculated from the temperature derivative of the electrons' total energy using the tight-binding model (*44*). The calculated temperature dependence of C_e is plotted in Fig. S14. $C_d = C_{l-Gr} + C_{l-MoS_2}$ is a combination of graphene and MoS_2 lattice heat capacity. Since the device is symmetric along out-of-plane direction, the model is based on top half of the stack, i.e., $C_{l-Gr}(C_{l-MoS_2})$ is the lattice heat capacity of monolayer graphene (MoS_2). C_{l-Gr} has linear temperature dependence around room temperature and is approximately three thousand times larger than C_e at around room temperature (*45*). C_{l-MoS_2} is obtained from reference 46. Q_{IR} is the optical energy absorbed by a monolayer graphene from the IR-pulse, which is approximately 2% of the pulse energy. G_{G-hBN} = $55\,\mathrm{MW\,m^{-2}\,K^{-1}}$ is the thermal conductivity between graphene and hBN. Since the VIS-pulse is much weaker than the IR-pulse, the pulse heating effect is neglected. As a result, we can quantitatively track the evolution of the transient temperature of the device. By incorporating the experimental condition of the pump-probe photocurrent measurement in Fig. 3b, we obtain a T_d maximum of approximately 330 K after IR-pulse excitation. The photocurrent modelling is carried out in the time domain with the cross-correlation relation and a time-dependent transient photocurrent profile as presented in the main text.

REFERENCES AND NOTES

1. J. Sung, Y. Zhou, G. Scuri, V. Zólyomi, T. I. Andersen, H. Yoo, D. S. Wild, A. Y. Joe, R. J. Gelly, H. Heo, S. J. Magorrian, D. Bèrubè, A. M. M. Valdivia, T. Taniguchi, K. Watanabe, M. D. Lukin, P. Kim, V. I. Fal'ko, H. Park, Broken mirror symmetry in excitonic response of reconstructed domains in twisted $MoSe_2$/$MoSe_2$ bilayers. *Nat. Nanotechnol.* **15**, 750–754 (2020).

2. X. Wang, K. Yasuda, Y. Zhang, S. Liu, K. Watanabe, T. Taniguchi, J. Hone, L. Fu, P. Jarillo-Herrero, Interfacial ferroelectricity in rhombohedral-stacked bilayer transition metal dichalcogenides. *Nat. Nanotechnol.* **17**, 367–371 (2022).

3. M. Vizner Stern, Y. Waschitz, W. Cao, I. Nevo, K. Watanabe, T. Taniguchi, E. Sela, M. Urbakh, O. Hod, M. Ben Shalom, Interfacial ferroelectricity by van der Waals sliding. *Science* **372**, 1462–1466 (2021).

4. M. Wu, J. Li, Sliding ferroelectricity in 2D van der Waals materials: Related physics and future opportunities. *Proc. Natl. Acad. Sci. U.S.A.* **118**, e2115703118 (2021).

5. A. Weston, E. G. Castanon, V. Enaldiev, F. Ferreira, S. Bhattacharjee, S. Xu, H. Corte-León, Z. Wu, N. Clark, A. Summerfield, T. Hashimoto, Y. Gao, W. Wang, M. Hamer, H. Read, L. Fumagalli, A. V. Kretinin, S. J. Haigh, O. Kazakova, A. K. Geim, V. I. Fal'ko, R. Gorbachev, Interfacial ferroelectricity in marginally twisted 2D semiconductors. *Nat. Nanotechnol.* **17**, 390–395 (2022).

6. J. Liang, D. Yang, J. Wu, J. I. Dadap, K. Watanabe, T. Taniguchi, Z. Ye, Optically probing the asymmetric interlayer coupling in rhombohedral-stacked MoS_2 bilayer. *Phys. Rev. X* **12**, 041005 (2022).

7. D. Yang, J. Wu, B. T. Zhou, J. Liang, T. Ideue, T. Siu, K. M. Awan, K. Watanabe, T. Taniguchi, Y. Iwasa, M. Franz, Z. Ye, Spontaneous-polarization-induced photovoltaic effect in rhombohedrally stacked MoS_2. *Nat. Photon.* **16**, 469–474 (2022).

8. T. Akamatsu, T. Ideue, L. Zhou, Y. Dong, S. Kitamura, M. Yoshii, D. Yang, M. Onga, Y. Nakagawa, K. Watanabe, T. Taniguchi, J. Laurienzo, J. Huang, Z. Ye, T. Morimoto, H. Yuan, Y. Iwasa, A van der Waals interface that creates in-plane polarization and a spontaneous photovoltaic effect. *Science* **372**, 68–72 (2021).

9. X. Hong, J. Kim, S.-F. Shi, Y. Zhang, C. Jin, Y. Sun, S. Tongay, J. Wu, Y. Zhang, F. Wang, Ultrafast charge transfer in atomically thin MoS_2/WS_2 heterostructures. *Nat. Nanotechnol.* **9**, 682–686 (2014).

10. L. Yuan, T.-F. Chung, A. Kuc, Y. Wan, Y. Xu, Y. P. Chen, T. Heine, L. Huang, Photocarrier generation from interlayer charge-transfer transitions in WS_2-graphene heterostructures. *Sci. Adv.* **4**, e1700324 (2018).

11. X. Zhang, D. He, L. Yi, S. Zhao, J. He, Y. Wang, H. Zhao, Electron dynamics in MoS_2-graphite heterostructures. *Nanoscale* **9**, 14533–14539 (2017).

12. D. Luo, J. Tang, X. Shen, F. Ji, J. Yang, S. Weathersby, M. E. Kozina, Z. Chen, J. Xiao, Y. Ye, T. Cao, G. Zhang, X. Wang, A. M. Lindenberg, Twist-angle-dependent ultrafast charge transfer in MoS_2-graphene van der Waals heterostructures. *Nano Lett.* **21**, 8051–8057 (2021).

13. Y. Chen, Y. Li, Y. Zhao, H. Zhou, H. Zhu, Highly efficient hot electron harvesting from graphene before electron-hole thermalization *Sci. Adv.* **5**, eaax9958 (2019).

14. C. Jin, E. Y. Ma, O. Karni, E. C. Regan, F. Wang, T. F. Heinz, Ultrafast dynamics in van der Waals heterostructures. *Nat. Nanotechnol.* **13**, 994–1003 (2018).

15. J.-Y. Wu, Y. T. Chun, S. Li, T. Zhang, J. Wang, P. K. Shrestha, D. Chu, Broadband MoS_2 field-effect phototransistors: Ultrasensitive visible-light photoresponse and negative infrared photoresponse. *Adv. Mater.* **30**, 1705880 (2018).

16. W. Zhang, C.-P. Chuu, J.-K. Huang, C.-H. Chen, M.-L. Tsai, Y.-H. Chang, C.-T. Liang, Y.-Z. Chen, Y.-L. Chueh, J.-H. He, M.-Y. Chou, L.-J. Li, Ultrahigh-gain photodetectors based on atomically thin graphene-MoS_2 heterostructures. *Sci. Rep.* **4**, 3826 (2014).

17. M. Buscema, M. Barkelid, V. Zwiller, H. S. van der Zant, G. A. Steele, A. Castellanos-Gomez, Large and tunable photothermoelectric effect in single-layer MoS_2. *Nano Lett.* **13**, 358–363 (2013).

18. D. J. Groenendijk, M. Buscema, G. A. Steele, S. Michaelis de Vasconcellos, R. Bratschitsch, H. S. J. van der Zant, A. Castellanos-Gomez, Photovoltaic and photothermoelectric effect in a double-gated WSe_2 device. *Nano Lett.* **14**, 5846–5852 (2014).

19. N. M. Gabor, J. C. Song, Q. Ma, N. L. Nair, T. Taychatanapat, K. Watanabe, T. Taniguchi, L. S. Levitov, P. Jarillo-Herrero, Hot carrier–assisted intrinsic photoresponse in graphene. *Science* **334**, 648–652 (2011).

20. M. Freitag, T. Low, F. Xia, P. Avouris, Photoconductivity of biased graphene. *Nat. Photon.* **7**, 53–59 (2013).

21. W. J. Yu, Q. A. Vu, H. Oh, H. G. Nam, H. Zhou, S. Cha, J.-Y. Kim, A. Carvalho, M. Jeong, H. Choi, A. H. C. Neto, Y. H. Lee, X. Duan, Unusually efficient photocurrent extraction in monolayer van der Waals heterostructure by tunnelling through discretized barriers. *Nat. Commun.* **7**, 13278 (2016).

22. L. Hou, Q. Zhang, M. Tweedie, V. Shautsova, Y. Sheng, Y. Zhou, H. Huang, T. Chen, J. H. Warner, Photocurrent direction control and increased photovoltaic effects in all-2D ultrathin vertical heterostructures using asymmetric h-BN tunneling barriers. *ACS Appl. Mater. Interfaces* **11**, 40274–40282 (2019).

23. D. Daranciang, M. J. Highland, H. Wen, S. M. Young, N. C. Brandt, H. Y. Hwang, M. Vattilana, M. Nicoul, F. Quirin, J. Goodfellow, T. Qi, I. Grinberg, D. M. Fritz, M. Cammarata, D. Zhu, H. T. Lemke, D. .A. Walko, E. M. Dufresne, Y. Li, J. Larsson, D. A. Reis, K. Sokolowski-Tinten, K. A. Nelson, A. M. Rappe, P. H. Fuoss, G. B. Stephenson, A. M. Lindenberg, Ultrafast photovoltaic response in ferroelectric nanolayers. *Phys. Rev. Lett.* **108**, 087601 (2012).

24. M. Massicotte, P. Schmidt, F. Vialla, K. G. Schädler, A. Reserbat-Plantey, K. Watanabe, T. Taniguchi, K. J. Tielrooij, F. H. L. Koppens, Picosecond photoresponse in van der Waals heterostructures. *Nat. Nanotechnol.* **11**, 42–46 (2016).

25. M. Massicotte, P. Schmidt, F. Vialla, K. Watanabe, T. Taniguchi, K. J. Tielrooij, F. H. L. Koppens, Photo-thermionic effect in vertical graphene heterostructures. *Nat. Commun.* **7**, 12174 (2016).

26. D. Sun, G. Aivazian, A. M. Jones, J. S. Ross, W. Yao, D. Cobden, X. Xu, Ultrafast hot-carrier-dominated photocurrent in graphene. *Nat. Nanotechnol.* **7**, 114–118 (2012).

27. M. W. Graham, S.-F. Shi, D. C. Ralph, J. Park, P. L. McEuen, Photocurrent measurements of supercollision cooling in graphene. *Nat. Phys.* **9**, 103–108 (2013).

28. A. C. Betz, S. H. Jhang, E. Pallecchi, R. Ferreira, G. Fève, J. M. Berroir, B. Plaçais, Supercollision cooling in undoped graphene. *Nat. Phys.* **9**, 109–112 (2013).

29. Z. Nie, R. Long, L. Sun, C.-C. Huang, J. Zhang, Q. Xiong, D. W. Hewak, Z. Shen, O. V. Prezhdo, Z.-H. Loh, Ultrafast carrier thermalization and cooling dynamics in few-layer MoS_2. *ACS Nano* **8**, 10931–10940 (2014).

30. K. T. Vogt, S.-F. Shi, F. Wang, M. W. Graham, Ultrafast photocurrent and absorption microscopy of few-layer transition metal dichalcogenide devices that isolate rate-limiting dynamics driving fast and efficient photoresponse. *J. Phys. Chem. C* **124**, 15195–15204 (2020).

31. D. Sun, Y. Rao, G. A. Reider, G. Chen, Y. You, L. Brézin, A. R. Harutyunyan, T. F. Heinz, Observation of rapid exciton–exciton annihilation in monolayer molybdenum disulfide. *Nano Lett.* **14**, 5625–5629 (2014).

32. H. Wang, C. Zhang, F. Rana, Ultrafast dynamics of defect-assisted electron–hole recombination in monolayer MoS_2. *Nano Lett.* **15**, 339–345 (2015).

33. T. Georgiou, R. Jalil, B. D. Belle, L. Britnell, R. V. Gorbachev, S. V. Morozov, Y.-J. Kim, A. Gholinia, S. J. Haigh, O. Makarovsky, L. Eaves, L. A. Ponomarenko, A. K. Geim, K. S. Novoselov, A. Mishchenko, Vertical field-effect transistor based on graphene–WS2 heterostructures for flexible and transparent electronics. *Nat. Nanotechnol.* **8**, 100–103 (2013).

34. K. Yasuda, X. Wang, K. Watanabe, T. Taniguchi, P. Jarillo-Herrero, Stacking-engineered ferroelectricity in bilayer boron nitride. *Science* **372**, 1458–1462 (2021).

35. Y. Liu, S. Liu, B. Li, W. J. Yoo, J. Hone, Identifying the transition order in an artificial ferroelectric van der Waals heterostructure. *Nano Lett.* **22**, 1265–1269 (2022).

36. Y. Liu, Z.-Y. Ong, J. Wu, Y. Zhao, K. Watanabe, T. Taniguchi, D. Chi, G. Zhang, J. T. Thong, C.-W. Qiu, K. Hippalgaonkar, Thermal conductance of the 2D MoS_2/*h*-BN and graphene/*h*-BN interfaces. *Sci. Rep.* **7**, 1–8 (2017).

37. A. A. Balandin, Thermal properties of graphene and nanostructured carbon materials. *Nat. Mater.* **10**, 569–581 (2011).

38. J. C. Song, M. Y. Reizer, L. S. Levitov, Disorder-assisted electron-phonon scattering and cooling pathways in graphene. *Phys. Rev. Lett.* **109**, 106602 (2012).

39. M. M. Furchi, D. K. Polyushkin, A. Pospischil, T. Mueller, Mechanisms of photoconductivity in atomically thin MoS_2. *Nano Lett.* **14**, 6165–6170 (2014).

40. C.-H. Lee, G.-H. Lee, A. M. Van Der Zande, W. Chen, Y. Li, M. Han, X. Cui, G. Arefe, C. Nuckolls, T. F. Heinz, J. Guo, J. Hone, P. Kim, Atomically thin p–n junctions with van der Waals heterointerfaces. *Nat. Nanotechnol.* **9**, 676–681 (2014).

41. E. Y. Ma, B. Guzelturk, G. Li, L. Cao, Z.-X. Shen, A. M. Lindenberg, T. F. Heinz, Recording interfacial currents on the subnanometer length and femtosecond time scale by terahertz emission. *Sci. Adv.* **5**, eaau0073 (2019).

42. M. Zhao, Z. Ye, R. Suzuki, Y. Ye, H. Zhu, J. Xiao, Y. Wang, Y. Iwasa, X. Zhang, Atomically phase-matched second-harmonic generation in a 2D crystal. *Light Sci. Appl.* **5**, e16131 (2016).

43. S. M. Sze, Y. Li, K. K. Ng, *Physics of Semiconductor Devices* (John Wiley & Sons, 2021).

44. D. Moldovan, M. Andelkovic, F. Peeters, pybinding v0.9.5: A Python package for tight-binding calculations (2020).

45. E. Pop, V. Varshney, A. K. Roy, Thermal properties of graphene: Fundamentals and applications. *MRS Bull.* **37**, 1273–1281 (2012).

46. D. Saha, S. Mahapatra, Analytical insight into the lattice thermal conductivity and heat capacity of monolayer MoS_2. *Phys. E* **83**, 455–460 (2016).
